# Characterization of Retinal Drusen in Subjects at High Genetic Risk of Developing Sporadic Alzheimer’s Disease: An Exploratory Analysis

**DOI:** 10.3390/jpm12050847

**Published:** 2022-05-23

**Authors:** Inés López-Cuenca, Elena Salobrar-García, Inés Gil-Salgado, Lidia Sánchez-Puebla, Lorena Elvira-Hurtado, José A. Fernández-Albarral, Federico Ramírez-Toraño, Ana Barabash, Jaisalmer de Frutos-Lucas, Juan J. Salazar, José M. Ramírez, Ana I. Ramírez, Rosa de Hoz

**Affiliations:** 1 Ramon Castroviejo Institute of Ophthalmologic Research, Group UCM 920105, Health Research Institute of the Hospital Clínico San Carlos (IdISSC), Complutense University of Madrid, 28040 Madrid, Spain; inelopez@ucm.es (I.L.-C.); elenasalobrar@med.ucm.es (E.S.-G.); inegil01@ucm.es (I.G.-S.); lidsan02@ucm.es (L.S.-P.); marelvir@ucm.es (L.E.-H.); joseaf08@ucm.es (J.A.F.-A.); jjsalazar@med.ucm.es (J.J.S.); ramirezs@med.ucm.es (J.M.R.); airamirez@med.ucm.es (A.I.R.); 2 Department of Immunology, Ophthalmology and ENT, Faculty of Optics and Optometry, Complutense University of Madrid, 28037 Madrid, Spain; 3 Laboratory of Cognitive and Computational Neuroscience, Center for Biomedical Technology, Technical University of Madrid, 28233 Madrid, Spain; federami@ucm.es (F.R.-T.); jaisamer.defrutos@ctb.upm.es (J.d.F.-L.); 4 Department of Experimental Psychology, Universidad Complutense de Madrid, 28223 Madrid, Spain; 5 Department of Endocrinology and Nutrition, IdISSC, 28040 Madrid, Spain; ana.barabash@gmail.com; 6 Diabetes and Associated Metabolic Diseases Networking Biomedical Research Centre, Carlos III Health Institute, 28029 Madrid, Spain; 7 Department of Medicine II, School of Medicine, Complutense University of Madrid, 28040 Madrid, Spain; 8 Centre for Precision Health, Edith Cowan University, Joondalup, WA 6027, Australia; 9 Department of Psychology, Faculty of Life and Nature Sciences, Antonio de Nebrija University, 28015 Madrid, Spain; 10 Department of Immunology, Ophthalmology and ENT, School of Medicine, Complutense University of Madrid, 28040 Madrid, Spain

**Keywords:** Alzheimer’s disease, ApoE ɛ4, family history, hard drusen, OCT, retina, AMD, hypercholesterolemia, hypertension, diabetes mellitus, choroid

## Abstract

Having a family history (FH+) of Alzheimer’s disease (AD) and being a carrier of at least one ɛ4 allele of the ApoE gene are two of the main risk factors for the development of AD. AD and age-related macular degeneration (AMD) share one of the main risk factors, such as age, and characteristics including the presence of deposits (Aβ plaques in AD and drusen in AMD); however, the role of apolipoprotein E isoforms in both pathologies is controversial. We analyzed and characterized retinal drusen by optical coherence tomography (OCT) in subjects, classifying them by their AD FH (FH- or FH+) and their allelic characterization of ApoE ɛ4 (ApoE ɛ4- or ApoE ɛ4+) and considering cardiovascular risk factors (hypercholesterolemia, hypertension, and diabetes mellitus). In addition, we analyzed the choroidal thickness by OCT and the area of the foveal avascular zone with OCTA. We did not find a relationship between a family history of AD or any of the ApoE isoforms and the presence or absence of drusen. Subjects with drusen show choroidal thinning compared to patients without drusen, and thinning could trigger changes in choroidal perfusion that may give rise to the deposits that generate drusen.

## 1. Introduction

Alzheimer’s disease (AD) is a neurodegenerative disease characterized by cognitive and functional impairment, in which age and genetic predisposition are two of the most important risk factors for its development [1]. One of the pathological features is the formation of extracellular beta-amyloid (Aβ) plaques and intracellular neurofibrillary tangles in the brains of AD patients [2].

Age-related macular degeneration (AMD) is retinal neurodegeneration that is one of the leading causes of visual impairment and blindness worldwide [3], and it is characterized by abnormal extracellular deposits known as drusen. Hard phenotypes of such deposits are common in older individuals [4] and do not represent a significant risk for developing this eye disease [5].

The prevalence of Alzheimer’s increases significantly between the ages of 65 and 85 [6]; coincidentally, AMD is the leading cause of visual impairment after the age of 65 in developed countries [7].

Although both pathologies share one of the main risk factors such as age and features including the presence of deposits (Aβ plaques in AD and waste substances in AMD) [8], genes such as apolipoprotein E (ApoE) seem to present opposite behaviors in the development of these pathologies. The ApoE gene, which has been implicated in modulating the metabolism and aggregation of Aβ [9], seems to have completely different responses in AD and AMD. This multifunction protein has three isoforms: ɛ2, ɛ3, and ɛ4. Thus, while the ApoE ɛ4 allele increases the risk of developing an AD [10], it appears to confer on its carriers a protective effect for the development of AMD [11]. This protective factor seems to act only in the advanced and wet forms of this retinal degeneration [12]. On the contrary, other authors did not find this protective effect for the ɛ4 allele, even suggesting that the presence of this allele is a risk factor for the development of AMD [13].

The ApoE ɛ2 allele has been associated with a slightly increased risk of developing late AMD, and an increased risk of disease progression when compared with ɛ3 carriers [14,15]. However, in a meta-analysis, there is no evidence to support the relationship between ApoE ɛ2 and AMD [16], and another recent study shows that the ɛ2 isoform has a protective role in wet forms of the disease [17].

Regarding the ɛ3 allele, it has been shown in the ApoE ɛ3 Leiden (E3L) mouse model that it has a hyperlipidemic phenotype with a prominent increase in very-low-density lipoprotein (VLDL) and low-density lipoprotein (LDL) [18], being more sensitive to lipid-lowering drugs than ApoE-/- and LDLr-/- mice [19]. In addition, it has previously been shown that hyperlipidemia alone can cause structural changes in the choroidal and retinal vascular system, which may eventually lead to retinal dysfunction [20]. In fact, in previous work, we have shown that the presence of hard drusen (HD) in subjects without ApoE ɛ4 carriers and without a family history of AD causes a statistically significant reduction in choroidal thickness when compared with cognitively healthy subject carriers of ApoE ɛ4 with FH+ [21]. This reduction in choroidal thickness could mean a reduction in blood flow, which is also found in pathologies such as AMD, glaucoma, or diabetic retinopathy [22,23,24].

The importance of cardiovascular risk factors in the AD onset, including hypertension, hypercholesterolemia, diabetes mellitus [25], obesity, compromised cardiac function, cerebral arterial disease, and physical inactivity, is now well-known [26]. These comorbidities cause thickening and loss of elasticity of the arterial wall and stiffening of the arterial intima leading to lipid accumulation in the artery [27]. In addition, it has been suggested that retinal lesions may reflect persistent small vessel damage due to hypertension and possibly inflammation and endothelial dysfunction [24].

Although some studies have classified drusen according to their morphology or according to their content in relation to other neurodegenerative diseases [4,28,29], to our knowledge, this is the first study that makes a morphological classification of drusen that appears in subjects with high genetic risk of developing AD, i.e., who are carriers of at least one ɛ4 allele for the ApoE gene and have a family member affected by sporadic AD, classifying them by the presence of comorbidities such as hypercholesterolemia, arterial hypertension, and diabetes.

## 2. Materials and Methods

### 2.1. Study Design

As described in previous works [21,30], this study is part of the project entitled “The cognitive and neurophysiological characteristics of subjects at high risk of developing dementia: a multidimensional approach” (COGDEM study).

Written informed consent was signed by all participants, which followed the principles of the Declaration of Helsinki. The local Ethics Committee of Hospital Clínico San Carlos approved this study with the internal code 18/422-E_BS.

**Figure 1:**
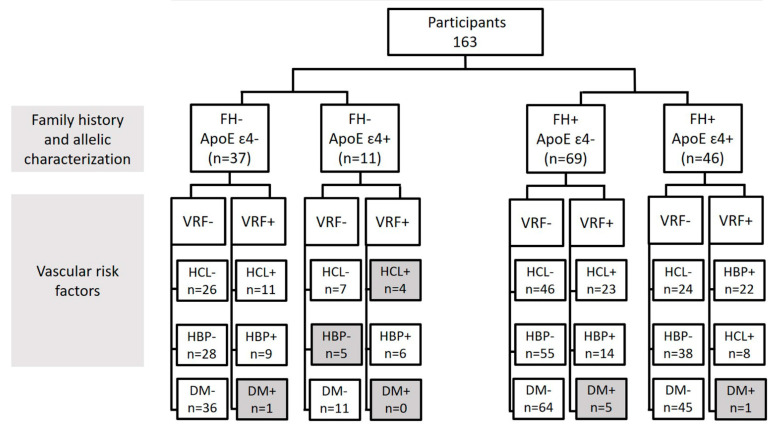
Flow chart of the subjects included in the study according to the different characteristics. (FH, family history; ApoE, apolipoprotein E; VRF, vascular risk factors, HCL, hypercholesterolemia, HBP, high blood pressure; and DM, diabetes mellitus). In gray, the groups discarded for having an n < 6 are shown.

The classification of the subjects is detailed in Figure 1. Firstly, we classified the participants into two study groups: those subjects who had no history of AD (FH-) and those who had at least one parent affected by the disease (FH+) (Figure 1).

Secondly, groups were subdivided according to the genotype for the ApoE gene (ApoE ɛ4- and ApoE ɛ4+). In addition, we analyzed the groups, taking into account both alleles to the ApoE gene, with the following groups: ApoE ɛ2ɛ2, ApoE ɛ2ɛ3, ApoE ɛ2ɛ4, ApoE ɛ3ɛ3, ApoE ɛ3ɛ4, and ApoE ɛ4ɛ4 (Figure 1).

Finally, it was further subdivided by also taking into account the vascular risk factors (hypercholesterolemia (HCL), high blood pressure (HBP), and diabetes) (Figure 1). Information on the cardiovascular status of the patients was extracted from a complete anamnesis. Participants were asked if they had and were being treated for HCL, HBP, or diabetes. Due to the detailed classification of the participants, no statistics were performed for groups with fewer than six subjects.

All subjects had a normal score on the Mini-Mental State Examination (MMSE) (above 26), no evidence of brain lesion or pathology, and a normal magnetic resonance image (MRI).

### 2.2. Ophthalmological Examination

Participants were scheduled by telephone to perform a complete ophthalmological examination in the clinic of the IIORC. During this call, they were screened to check their ophthalmological status. This included questions such as whether they wear glasses and if they know their approximate prescription, if they were under ophthalmic treatment, or if they had had previous eye surgeries. The visual examination included refraction, visual acuity measurement, biomicroscopy, intraocular pressure (IOP) and retinal analysis by optical coherence tomography (OCT), and OCT angiography (OCTA).

Ophthalmological inclusion criteria included a refraction less of ±5 spherocylindrical diopters, visual acuity > 0.5 dec, IOP < 21 mmHg. In addition, the participants were free of retinal pathology (as hypertensive or diabetic retinopathy) or congenital ocular malformations and did not have glaucoma or were not suspected of having it.

### 2.3. Drusen Characterization by OCT

OCT images were acquired with a Spectralis OCT (Heidelberg Engineering, Heidelberg, Germany). To be included in the study, scans had to be of high quality with a minimum signal-to-noise ratio of 25 and a mean of 16 B-scans.

**Figure 2:**
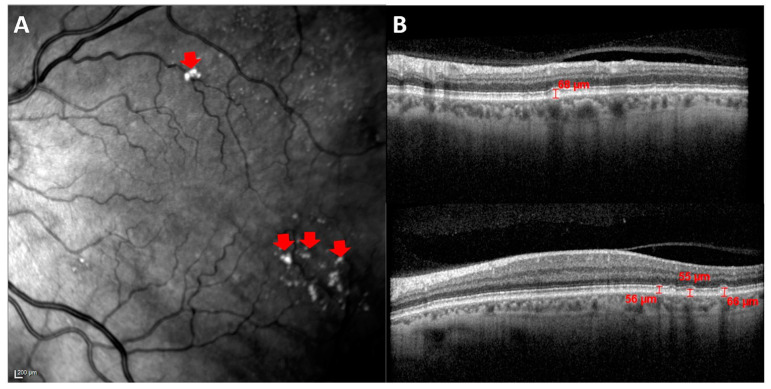
Analysis of drusen by OCT. (**A**) HRA fundus image. The red arrows show hyperreflective shapes. (**B**) Cross-sectional OCT scans showing drusen measurements in µm. This accumulation of hyperreflective material is located between the basal lamina of the RPE and the inner collagen layer of Bruch’s membrane.

In these images, drusen were identified as hyperreflective shapes on high reflectance fundus images (HRA) (Figure 2A) and as hyperreflective material located between the basal lamina of the retinal pigment epithelium (RPE) and the inner collagen layer of Bruch’s membrane on cross-sectional OCT scans.

The drusen measurement was carried out with the OCT software. As can be seen in Figure 2B, a vertical line was drawn between the basal lamina of the EPR and the highest point of the drusen cupule, which coincides with the inner collagen layer of Bruch’s membrane.

The classification of these drusen was carried out based on the guidelines of the Protocol for diagnosis, follow-up, and general recommendations in early and intermediate age-related degeneration (AMD): consensus of a panel of experts [31]. Following these guidelines, drusen can be classified according to their arrangement, location, size, and type. A summary of the criteria for the classification of drusen is given in Table 1.

In all participants we counted the total number of drusen, and the mean measurement was taken as the average of the measurement of different drusen up to a maximum of 10.

We also analyzed the choroidal thickness at 10 points around the fovea with OCT and the foveal avascular zone (FAZ) with OCTA. The choroidal thickness was manually delimited from the outer hyperreflective line to the sclerochoroidal interface of the RPE; for the FAZ, it was also delimited manually. These procedures have been developed and explained in detail in previous works [21,32].

The classification and measurement of drusen, choroidal thickness, and FAZ were performed by the same trained researcher, who is blind to the subject FH or genotype to avoid influence in the measurements.

The colorimetric representation of the choroidal thickness between study groups was created with the color scale function in Microsoft Excel. The values were normalized for this scale. Value 1, in white, was where there was no difference, −0.5 in the blue tone, for thinning of the choroid, and 1.5 with the red tone, when the choroid was thickened. The software provides the color tone directly according to the thickness variation.

### 2.4. ApoE Genotyping

Genomic DNA was extracted from whole blood in EDTA using standard DNA isolation methods (DNAzol^®^; Molecular Research Center, Inc., Cincinnati, OH, USA) from FH+ and FH- subjects. Two single nucleotide polymorphisms (SNPs), rs7412 and rs429358, were genotyped using TaqMan genotyping assays on an Applied Biosystems 7500 rapid real-time PCR instrument (Applied Biosystems, Forster City, CA, USA). Accordingly, ApoE haplotypes were established. Negative sample controls and sample controls for each genotype were included in each assay. Several intra- and interplate duplicates of DNA samples were included

### 2.5. Statistical Analysis

Statistical analysis was performed using SPSS 27.0 (SPSS Inc., Inc., Chicago, IL, USA). The differences between study groups were analyzed using the Mann–Whitney U test. Data are expressed as mean ± standard deviation (SD). A *p* value < 0.05 was considered statistically significant.

## 3. Results

### 3.1. Demographic Data

The patients were aged between 45 and 80, were Caucasic, and had a mean MMSE score of 29.00 ± 0.73.

Regarding the drusen type, none of the patients presented soft drusen, and only hard drusen were present in the retina of these patients. No patient presented large drusen (>125 µm).

Subgroups with n < 6 were discarded from the statistical study.

### 3.2. Characterization of Drusen by Family History

In the FH- group, 63.79% of participants presented hard drusen, while in the FH+ group it was 61.53%. Characteristics of distribution, location, and size of drusen can be observed in Table 2.

When we compared the drusen number and size between groups, we found no statistically significant differences (*p* > 0.05) (Table 2).

### 3.3. Characterization of Drusen by ApoE Genotype

The presence of hard drusen in the ApoE ɛ4- group was detected in 61.53% of the subjects and in the ApoE ɛ4+ group in 62.07%. There is no difference in drusen number and size between groups. Details of drusen distribution, location, number, and size can be found in Table 2.

When we analyzed the two alleles for the ApoE gene, we classified the subjects into the following groups: ApoE ɛ2ɛ2 (n = 1), ApoE ɛ2ɛ3 (*n* = 9), ApoE ɛ2ɛ4 (*n* = 2), ApoE ɛ3ɛ3 (*n* = 107), ApoE ɛ3ɛ4 (*n* = 51), and ApoE ɛ4ɛ4 (*n* = 6).

The presence of drusen according to the allelic characterization was: (i) 64.48% in ApoE ɛ3ɛ3 and (ii) 62.74% in ApoE ɛ3ɛ4. Details of drusen distribution, location, number, and size can be found in Table 3.

When we compared the mean number and size of drusen between the ApoE ɛ3ɛ3 vs. ApoE ɛ3ɛ4 groups, we found no statistically significant differences (*p* > 0.05) (Table 3).

### 3.4. Characterization of Drusen by Family History and ApoE Genotype

No statistically significant differences (*p* > 0.05) were found in the mean number of drusen or the mean size of drusen when compared between the study groups (Supplementary Materials Table S1).

### 3.5. Characterization of Drusen by Family History, ApoE Genotype, and Vascular Risk Factors

Taking into account the classification of FH and ApoE genotype, the following vascular risk factors were analyzed: (i) HCL; (ii) HPB; (iii) diabetes mellitus.

#### 3.5.1. Hypercholesterolemia (HCL)

We found no statistically significant differences (*p* > 0.05) in the presence, distribution, and location of drusen within these study groups (Table 4).

Although there were no significant differences, the group with the greatest drusen number was FH- ApoE ɛ4+ HCL- (71.43%), followed by FH- ApoE ɛ4- HCL- (65.38%), and the group with the lowest percentage of drusen was FH- ApoE ɛ4- HCL+, with 45.45% (Table 4).

In addition, when we compared the drusen number between groups, there was statistical significance (*p* < 0.05) between the FH- ApoE ɛ4- HCL+ group (12.80 ± 11.99) and (i) FH+ ApoE ɛ4- HCL+ (4.60 ± 5.03) (*p*-value = 0.038) and (ii) FH+ ApoE ɛ4+ HCL- (4.67 ± 3.44) (*p*-value = 0.025) (Table 5). When we compared the mean drusen size between groups, we found no statistically significant differences (Table 5).

**Figure 3:**
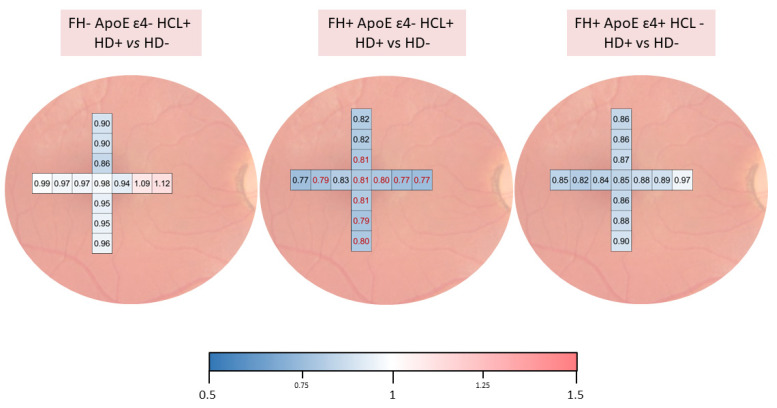
Colorimetric representation of choroidal thickness analysis at different points between participants with and without drusen in FH-ApoE ɛ4- HCL+, FH+ ApoE ɛ4- HCL+, and FH+ ApoE ɛ4+ HCL-. Thinning is shown in shades of blue, while thickening is shown in red tones.

In these study groups, we had analyzed choroidal thickness and found statistically significant differences (*p* < 0.05) between FH+ ApoE ɛ4- HCL+ with hard drusen (HD+) and FH+ ApoE ɛ4- HCL+ without hard drusen (HD-) at different points of the choroid. Details can be seen in Figure 3.

When we analyzed the FAZ, both superficial and deep plexus, we found no statistically significant differences.

#### 3.5.2. Hypertension

The characteristics of the study groups are shown in Table 6.

There were no significant differences between drusen presence distribution and location between groups. The groups with a greater number of subjects with drusen were FH+ ApoE ɛ4+ HBP+ (75%), and FH+ ApoE ɛ4- HBP+ (71.43%) and the group with less presence of drusen was FH- ApoE ɛ4- HBP- (47.22%) (Table 6).

Comparing the drusen number between the study groups, we found statistically significant differences when comparing the FH- ApoE ɛ4- HBP- group (5.59 ± 3.69) and the FH+ ApoE ɛ4+ HBP+ group (2.67 ± 1.37) (*p* = 0.041). We also found statistically significant differences in the number of drusen when comparing the FH- ApoE ɛ4- HBP+ group (17.80 ± 15.00) with: (i) FH+ ApoE ɛ4- HBP- group (12.24 ± 27.49) (*p*-value 0.040); and (ii) FH+ ApoE ɛ4+ HBP+ group (2.67 ± 1.37) (*p*-value 0.016) (Table 6 and Table 7). When we compared the drusen size between the study groups we found no statistically significant differences (*p* > 0.05) (Table 7).

#### 3.5.3. Diabetes Mellitus

The characteristics of the study groups are shown in Table 8. When we compared the groups in terms of size and number of drusen, no statistically significant differences were found (*p* > 0.05) (Supplementary Materials Table S2).

## 4. Discussion

In this study of subjects at high genetic risk of developing AD, we carried out a strict characterization of retinal drusen, considering different classifications in relation to the different risk factors of the subjects under study: family history of AD; genetic characterization for ApoE; and cardiovascular risk factors such as HCL, arterial hypertension, and diabetes mellitus.

The first thing to note in this study population is that none of the participants had soft drusen. This is due to the strict selection of the study population and the inclusion criteria of the study, in which the subjects should not present macular structural modifications. The presence of drusen and AMD has been associated with AD in previous studies [33,34]. This is because the pathogenesis of both chronic neurodegenerative disorders shows some striking similarities, such as their relationship with aging, their unknown etiology, and, more specifically, the presence of senile plaques (extracellular with an inner core of Aβ peptide fibers) in both the cerebral grey matter and the retina [35]. Drusen may contain a large number of Aβ structures, with diameters varying between 0.25 and 10 μm and highly organized concentric layers when viewed under an electron microscope, or none at all [28]. In addition, Aβ is involved in complement activation in the drusen formation [5]. Aβ oligomers found in drusen are toxic to human retinal pigment epithelium and cultured SH-SY5Y human neuroblastoma cells [29], and these findings are consistent with studies in patients with early AD, where large amounts of these highly toxic oligomers are found in the brain, causing neuronal dysfunction and synaptic disruption [36]. This could explain the possible alterations found in both the retina and choroid of subjects at high genetic risk of developing AD, who are also part of this study, and which we have reported in a previous work [21,30]. We have also demonstrated these alterations in the retina of a murine model of preclinical AD [37]. All these findings support the idea that the changes produced by AD may appear early in the retina, even before the onset of brain alterations [38].

The high heritability of AD is well known [39], and first-degree family history is associated with an increased risk of developing this disease [40,41,42,43]. Although a relationship between AD and the presence of drusen has been reported [44,45], in the present study, there does not seem to be a relationship between family history of AD and the presence or absence of drusen. Furthermore, when we compared the number of drusen and their size between FH- and FH+, we found no significant differences.

A protective role of ApoE ɛ4 in relation to the development of AMD has been suggested. There are two hypotheses to explain this effect. The first is the absence of disulfide bridges in ApoE ɛ4, which makes it smaller in size and more easily transportable across Bruch’s membrane. The second hypothesis is that ApoE ɛ4 has a positive charge, which decreases the hydrophobicity of Bruch’s membrane and facilitates debris removal [46]. We found no statistically significant differences in the presence or absence of drusen, with respect to ApoE allelic characterization. However, our participants have hard drusen, and we do not know if they will develop AMD in the future, despite having at least one ɛ4 allele.

Non-genetic risk factors have been shown to play an important role in the development of AD, and it is likely that the interaction between genetic and environmental factors triggers the onset of pathophysiological events that ultimately lead to the development of this neurodegenerative disease [34]. There seems to be a relationship between the accumulation of cardiovascular risk factors, such as HCL, HBP, and diabetes, with age [47] and the risk of developing AD [25,26].

When analyzing the drusen’s features by family history, ApoE alleles, and the presence or absence of HCL, we found that there were statistically significant differences in the drusen number between the groups. In addition, when we studied the choroidal thickness, we found that participants with drusen have thinner choroids than subjects without drusen, which was statistically significant in the FH+ ApoE ɛ4- HCL+ HD+ group. This suggests that the presence of HCL along with the absence of ɛ4 in ApoE characterization could produce a decrease in choroidal thickness possibly caused by decreased perfusion and could ultimately lead to drusen formation. This may be because the increased risk conferred by the ɛ2 and, to a lesser extent, ɛ3 alleles, is compounded by the formation of atheroma plaques and accumulation of lipid deposits in the eye caused by high levels of VLDL in the blood. It is not known whether the formation of these drusen is caused by changes in the outer retina due to metabolic stress associated with fatty acid metabolism or from perfusion changes in the choroid due to arteriosclerosis [48]. Changes in Bruch’s membrane, the RPE, and retinal layers, as well as vascular alterations, would be responsible for chronic ischemia [7], which could increase the concentration of extracellular glutamate, leading to oxidative damage by a neuronal cytotoxic mechanism [49,50].

On the other hand, lipid deposits in a disrupted Bruch’s membrane create a hydrophobic barrier which obstructs the metabolic exchange between the choriocapillaris and the RPE [7,51]. This situation may contribute to the reduced supply of nutrients and oxygen to the retina, a situation that may play a pathogenic role in the development of AMD [52]. Previous studies have also shown that RPE cells secrete ApoE in response to various hormones and that it is related to HDL. This suggests a possible role for ApoE in AMD pathology related to retinal lipid trafficking [53]. Although the HCL subjects were treated pharmacologically, we know from previous studies that normalization of lipid levels is not followed by complete recovery of normal retinal histology [22] and that the remaining retinal changes are mainly due to chronic sustained ischemia caused by alterations in retinal vessels, Bruch’s membrane, and the RPE [51]. These ischemic situations have a detrimental impact on the retinal neurons [51].

In addition, we found no statistically significant differences in the FAZ in the different study groups. These findings support those presented in a previous work, where no alterations in retinal vascular flow were found in subjects at high genetic risk of developing AD [21]. In patients with established AD, but in early stages of AD, no changes were found in the FAZ or in the percentage of oxyhemoglobin measured in the optic nerve head, demonstrating that the choroidal vascular plexus is affected early, even preclinically, in AD and the retinal plexus is affected in advanced stages of the disease [32].

When we analyzed subjects in relation to HBP, we found that subjects of the FH- ApoE ɛ4- HBP- group had a higher number of drusen than the FH+ ApoE ɛ4+ HBP+ group. Thus, there does not appear to be a relationship between drusen and the blood pressure level of the participants, which again highlights the importance of the presence of ɛ2 and ɛ3 alleles with the presence of drusen in the retina. One possible explanation is that the study subjects had their blood pressure levels controlled by drug treatment and were, therefore, at normal levels.

We could not associate the presence of diabetes mellitus with the presence of drusen due to the small number of subjects with this pathology in our study groups.

The present work has limitations and strengths. It is the first study to characterize drusen in cognitively healthy participants with two well-defined risk factors for the development of AD. In addition, these patients are carefully classified based on family history, characterization for ApoE, and cardiovascular risk factors that may influence the development of retinal ischemia.

Because this is one of the first exploratory examinations in this healthy population at high genetic risk for the development of sporadic AD, we decided to be more flexible with the problem of multiple comparisons. We believe that these results may serve as a first step or guide for new hypotheses and future studies that validate our results and may reveal new biomarkers for AD.

## 5. Conclusions

In conclusion, there does not seem to be a relationship between FH of AD or any of the ApoE isoforms and the presence or absence of drusen. Subjects with drusen show choroidal thinning compared to patients without drusen, reaching statistical significance in the group of participants with an FH of AD, without ApoE ɛ4, and with HCL. This thinning could trigger changes in choroidal perfusion that may give rise to the deposits that generate drusen.

## Figures and Tables

**Table 1:** Classification of drusen according to the protocol of Ruiz et al. 2016.

Distribution	Location	Drusen Size	Drusen Type
Unilateral	Foveal: located below the fovea	Small (≤63 µm)	Hard (<125 µm)
Bilateral	Macular: located in the macular area between the vascular arcades	Median (>65 and ≤125 µm)	Soft (>125 µm)
	Peripheral: located outside the vascular arcades	Large (>125 µm)	
	Macular+peripheral: combination of the above locations		

**Table 2:** Characterization of drusen in subjects according to family history of AD and ApoE genotype.

Variables Analyzed	Study Groups	FH-	FH+	*p*-Value FH- vs. FH+	ApoE ɛ4-	ApoE ɛ4+	*p*-Value	FH- ApoE ɛ4-	FH- ApoE ɛ4+	FH+ ApoE ɛ4-	FH+ ApoE ɛ4+
		(*n* = 58)	(*n* = 130)		(*n* = 117)	(*n* = 58)	ApoE ɛ4- vs. ApoE ɛ4+	(*n* = 37)	(*n* = 11)	(*n* = 69)	(*n* = 46)
**Existence of drusen**	**YES**	37 (63.79%)	80 (61.54%)		72 (61.53%)	36 (62.07%)		22 (59.45%)	7 (63.64%)	44 (63.76%)	28 (60.87%)
	**NO**	21 (36.21%)	50 (38.46%)		45 (38.47%)	22 (37.95%)		15 (40.55%)	4 (36.36%)	25 (36.24%)	18 (39.13%)
**Distribution**	**Unilateral**	11 (29.73%)	41 (50.25%)		33 (45.83%)	15 (41.67%)		5 (22.73%)	1 (14.28%)	24 (54.55%)	13 (46.43%)
	**Bilateral**	26 (70.27%)	39 (48.75%)		39 (51.17%)	21 (58.33%)		17 (77.27%)	6 (85.72%)	20 (45.45%)	15 (53.57%)
**Location**	**Foveal**	0	1 (1.25%)		0	1 (2.77%)		0	0	0	1 (3.57%)
	**Macular**	5 (13.51%)	17 (21.25%)		13 (18.05%)	8 (22.22%)		2 (9.09%)	1 (14.28%)	10 (22.73%)	6 (21.43%)
	**Peripheral**	14 (37.83%)	28 (35.00%)		29 (40.27%)	8 (22.22%)		8 (36.36%)	1 (14.28%)	17 (38.64%)	7 (25.00%)
	**Macular + Peripheral**	18 (48.64%)	34 (42.50%)		30 (41.66%)	19 (52.77%)		12 (54.54%)	5 (71.42%)	17 (38.64%)	14 (50.00%)
**Drusen number ± SD**		10.86 ± 18.24	11.38 ± 25.14	0.077 ‡	9.99 ± 20.13	11.36 ± 22.28	0.950 ‡	8.36 ± 8.98	24.14 ± 37.79	11.55 ± 24.89	8.54 ± 16.48
**Drusen size ± SD (µm)**		59.98 ± 7.95	61.94 ± 11.43	0.783 ‡	61.08 ± 10.40	60.76 ± 11.05	0.764 ‡	58.42 ± 7.66	59.31 ± 6.22	62.00 ± 11.02	61.22 ± 12.15
**Drusen size classification**	**Small (≤63 µm)**	26 (70.27%)	52 (65.00%)		48 (66.66%)	26 (72.22%)		17 (77.27%)	5 (71.42%)	27 (61.36%)	20 (71.43%)
	**Medium (>65 and ≤125 µm)**	11 (29.73%)	18 (35.00%)		24 (33.33%)	10 (27.77%)		5 (22.72%)	2 (28.57%)	17 (38.64%)	8 (28.57%)

Mean ± SD; ‡ Mann–Whitney U Test, (FH, family history; ApoE, apolipoprotein E; SD, standard deviation).

**Table 3:** Characterization of drusen in subjects according to ApoE genotype.

Variables Analyzed	Study Groups	ApoE ɛ3ɛ3	ApoE ɛ3ɛ4	*p*-Value
		(*n* = 107)	(*n* = 51)	
**Existence of drusen**	**YES**	69 (64.48%)	32 (62.74%)	
	**NO**	38 (35.52%)	19 (37.26%)	
**Distribution**	**Unilateral**	32 (46.37%)	16 (50.00%)	
	**Bilateral**	37 (53.62%)	16 (50.00%)	
**Location**	**Foveal**	0	1 (3.13%)	
	**Macular**	13 (18.84%)	8 (25.00%)	
	**Peripheral**	27 (39.13%)	8 (25.00%)	
	**Macular + Peripheral**	29 (42.03%)	15 (46.88%)	
**Drusen number ± SD**		10.30 ± 20.51	10.94 ± 23.32	0.438
**Drusen size ± SD (µm)**		60.76 ± 10.32	61.77 ± 11.99	0.949
**Drusen size classification**	**Small (≤63 µm)**	47 (98.12%)	22 (68.75%)	
	**Medium (>65 and ≤125 µm)**	22 (31.88%)	10 (32.25%)	

Mean ± SD; (ApoE, apolipoprotein E; SD, standard deviation).

**Table 4:** Characterization of drusen by FH, ApoE genotype, and HCL.

		FH- ApoE ɛ4- HCL-	FH- ApoE ɛ4- HCL+	FH- ApoE ɛ4+ HCL-	FH+ ApoE ɛ4- HCL-	FH+ ApoE ɛ4- HCL+	FH+ ApoE ɛ4+ HCL-	FH+ ApoE ɛ4+ HCL+
		(*n* = 26)	(*n* = 11)	(*n* = 7)	(*n* = 46)	(*n* = 23)	(*n* = 24)	(*n* = 22)
**Existence of drusen**	**YES**	17 (65.38%)	5 (45.45%)	5 (71.43%)	29 (63.04%)	15 (65.22%)	15 (62.50%)	13 (59.09%)
	**NO**	9 (34.62%)	6 (54.55%)	2 (28.57%)	17 (36.96%)	8 (34.78%)	9 (37.5%)	9 (40.91%)
**Distribution**	**Unilateral**	5 (29.41%)	0	1 (20.00%)	15 (51.73%)	9 (60.00%)	8 (53.33%)	5 (38.46%)
	**Bilateral**	12 (70.59%)	5 (100%)	4 (80.00%)	14 (48.28%)	6 (40.00%)	7 (46.67%)	8 (61.54%)
**Location**	**Foveal**	0	0	0	0	0	0	1 (7.69%)
	**Macular**	1 (5.88%)	1 (20.00%)	1 (20.00%)	5 (17.24%)	5 (33.33%)	5 (33.33%)	1 (7.69%)
	**Peripheral**	8 (47.06%)	0	1 (20.00%)	12 (41.38%)	5 (33.33%)	4 (26.67%)	3 (23.08%)
	**Macular + Peripheral**	8 (47.06%)	4 (80.00%)	3 (60.00%)	12 (41.38%)	5 (33.33%)	6 (40.00%)	8 (61.54%)
**Drusen number ± SD**		12.53 ± 31.84	12.80 ± 11.99	24.60 ± 45.73	15.14 ± 30.00	4.60 ± 5.03	4.67 ± 3.44	13.00 ± 23.60
**Drusen size ± SD (µm)**		65.42 ± 10.74	61.59 ± 9.57	57.92 ± 7.30	61.73 ± 12.20	62.51 ± 8.64	59.05 ± 9.81	63.73 ± 14.38
**Drusen size classification**	**Small (≤63 µm)**	9 (52.94%)	3 (60.00%)	4 (80.00%)	19 (65.52%)	8 (53.33%)	12 (80.00%)	8 (61.54%)
	**Medium (>65 and ≤125 µm)**	8 (47.06%)	2 (40.00%)	1 (20.00%)	10 (34.48%)	7 (46.67%)	3 (20.00%)	5 (38.46%)

Mean ± SD; (FH, family history; ApoE: apolipoprotein E; HCL: hypercholesterolemia SD: standard deviation).

**Table 5:** *p*-value of drusen number and size between study groups based on family history, ApoE ɛ4- or ApoE ɛ4+ genotype, and hypercholesterolemia. Based on Mann–Whitney U Test.

		Drusen Size						
		FH- ApoE ɛ4- HCL-	FH- ApoE ɛ4- HCL+	FH- ApoE ɛ4+ HCL-	FH+ ApoE ɛ4- HCL-	FH+ ApoE ɛ4- HCL+	FH+ ApoE ɛ4+ HCL-	FH+ ApoE ɛ4+ HCL+
**Drusen number**	**FH- ApoE ɛ4- HCL-**		0.481	0.845	0.509	0.113	0.895	0.451
	**FH- ApoE ɛ4- HCL+**	0.070		0.465	0.903	0.600	0.631	0.921
	**FH- ApoE ɛ4+ HCL-**	0.723	0.463		0.808	0.238	0.662	0.522
	**FH+ ApoE ɛ4- HCL-**	0.731	0.096	0.922		0.480	0.683	0.817
	**FH+ ApoE ɛ4- HCL+**	0.143	0.038 *	0.559	0.255		0.300	0.678
	**FH+ ApoE ɛ4+ HCL-**	0.447	0.025 *	0.930	0.708	0.556		0.765
	**FH+ ApoE ɛ4+ HCL+**	0.758	0.092	0.881	0.934	0.362	0.626	

Mann–Whitney U Test, * *p* < 0.05.

**Table 6:** Characterization of drusen by FH, ApoE genotype, and HBP.

		FH- ApoE ɛ4- HBP-	FH- ApoE ɛ4- HBP+	FH- ApoE ɛ4+ HBP+	FH+ ApoE ɛ4- HBP-	FH+ ApoE ɛ4- HBP+	FH+ ApoE ɛ4+ HBP-	FH+ ApoE ɛ4+ HBP+
		(*n* = 28)	(*n* = 9)	(*n* = 6)	(*n* = 55)	(*n* = 14)	(*n* = 38)	(*n* = 8)
**Existence of drusen**	**YES**	17 (47.22%)	5 (55.55%)	4 (66.67%)	34 (61.82%)	10 (71.43%)	22 (57.89%)	6 (75%)
	**NO**	11 (52.78%)	4 (44.45%)	2 (33.33%)	21 (38.18%)	4 (28.57%)	16 (42.11%)	2 (25%)
**Distribution**	**Unilateral**	5 (29.41%)	0	1 (25.00%)	20 (58.82%)	4 (40.00%)	10 (45.45%)	3 (50.00%)
	**Bilateral**	12 (70.59%)	5 (100%)	3 (75.00%)	14 (41.17%)	6(60.00%)	12 (54.55%)	3 (50.00%)
**Location**	**Foveal**	0	0	0	0	0	0	1 (16.67%)
	**Macular**	1 (5.88%)	1 (20.00%)	1 (25.00%)	7 (20.59%)	3 (30.00%)	5 (22.73%)	1 (16.67%)
	**Peripheral**	8 (47.06%)	0	0	14 (41.18%)	3 (30.00%)	5 (22.73%)	2 (33.33%)
	**Macular + Peripheral**	8 (47.06%)	4 (80.00%)	3 (75.00%)	13 (38.24%)	4 (40.00%)	12 (54.55%)	2 (33.33%)
**Drusen number ± SD**		5.59 ± 3.69	17.80 ± 15.00	38.25 ± 47.06	12.24 ± 27.49	9.20 ± 13.44	10.14 ± 18.24	2.67 ± 1.37
**Drusen size ± SD (µm)**		58.42 ± 8.43	58.44 ± 4.89	61.15 ± 7.55	61.76 ± 12.24	62.81 ± 5.38	62.19 ± 12.34	57.68 ± 11.74
**Drusen size classification**	**Small (≤63 µm)**	13 (76.47%)	4 (80.88%)	2 (50.00%)	23 (67.65%)	4 (40.00%)	16 (72.73%)	4 (66.67%)
	**Medium (>65 and ≤125 µm)**	4 (23.53%)	1 (20.00%)	2 (50.00%)	11 (32.35%)	6 (60.00%)	6 (27.27%)	2 (33.33%)

Mean ± SD; (FH, family history; ApoE, apolipoprotein E; HCL, hypercholesterolemia; HBP, high blood pressure; SD, standard deviation).

**Table 7:** *p*-value of the number of drusen between the study groups according to FH, ApoE ɛ4- or ApoE ɛ4+ genotype, and HBP. Mann Whitney U test.

		Drusen Size					
		FH- ApoE ɛ4- HBP-	FH- ApoE ɛ4- HBP+	FH+ ApoE ɛ4- HBP-	FH+ ApoE ɛ4- HBP+	FH+ ApoE ɛ4+ HBP-	FH+ ApoE ɛ4+ HBP+
**Drusen number**	**FH- ApoE ɛ4- HBP-**		0.969	0,617	0.113	0.479	0.529
	**FH- ApoE ɛ4- HBP+**	0.054		0.867	0.086	0.901	0.584
	**FH+ ApoE ɛ4- HBP-**	0.268	0.040 *		0.287	0.775	0.363
	**FH+ ApoE ɛ4- HBP+**	0.820	0.139	0.543		0.329	0.233
	**FH+ ApoE ɛ4+ HBP-**	0.989	0.079	0.314	0.790		0.240
	**FH+ ApoE ɛ4+ HBP+**	0.041 *	0.016 *	0.420	0.249	0.080	

Mann–Whitney U Test, * *p* < 0.05.

**Table 8:** Characterization of drusen by family history, ApoE genotype, and diabetes mellitus.

		FH- ApoE ɛ4- DM-	FH- ApoE ɛ4+ DM-	FH+ ApoE ɛ4- DM-	FH+ ApoE ɛ4+ DM-
		(*n* = 36)	(*n* = 11)	(*n* = 64)	(*n* = 45)
**Existence of drusen**	**YES**	21 (58.33%)	7 (63.64%)	41 (64.06%)	27 (60.00%)
	**NO**	15 (41.67%)	4 (36.36%)	23 (35.94%)	18 (30%)
**Distribution**	**Unilateral**	5 (23.81%)	1 (14.29%)	22 (53.66%)	13 (48.15%)
	**Bilateral**	16 (76.19%)	6 (85.71%)	19 (46.34%)	14 (51.85%)
**Location**	**Foveal**	0	0	0	1 (3.70%)
	**Macular**	1 (4.76%)	1 (14.29%)	9 (21.95%)	6 (22.22%)
	**Peripheral**	8 (38.10%)	1 (14.29%)	16 (39.02%)	7 (25.93%)
	**Macular + Peripheral**	12 (57.14%)	5 (71,43%)	16 (39.02%)	13 (48.15%)
**Drusen number ± SD**		7.14 ± 7.09	24.14 ± 37.78	12.10 ± 25.70	8.63 ± 16.79
**Drusen size ± SD (µm)**		58.34 ± 7.84	59.31 ± 6.62	61.89 ± 11.30	60.83 ± 12.20
**Drusen size classification**	**Small (≤63 µm)**	16 (76.19%)	5 (71.43%)	26 (63,41%)	20 (74.07%)
	**Medium (>65 and ≤125 µm)**	5 (23.81%)	2 (28.57%)	15 (36.59%)	7 (25.93%)

Mean ± SD; (FH, family history; ApoE, apolipoprotein E; DM, diabetes mellitus; SD, standard deviation).

## Data Availability

The data supporting the findings of this study are available from the corresponding author upon request.

## Supplementary Materials

The following supporting information can be downloaded at: https://www.mdpi.com/article/10.3390/jpm12050847/s1, Table S1: *p*-value of drusen number and size groups characterized by history family and ApoE ɛ4 genotype. Mann–Whitney U test; Table S2: *p*-value of drusen number and size groups characterized by history family, ApoE ɛ4 genotype, and diabetes mellitus. Mann–Whitney U test.
