# Supplementary material for: Characterization of Retinal Drusen in Subjects at High Genetic Risk of Developing Sporadic Alzheimer’s Disease: An Exploratory Analysis
Source: J Pers Med. 2022 May 23;12(5):847. doi: 10.3390/jpm12050847 (PMC9145327; doi:10.3390/jpm12050847)
Supplement: Supplementary file 1 [file jpm-12-00847-s001.zip › jpm-1711892-supplementary.pdf]

Table S1. P- value of drusen number and size groups characterized by history family and ApoE ε4 genotype. Mann Whitney U test.

		Drusen size			
		**FH- ApoE ε4-**	**FH- ApoE ε4+**	**FH+ ApoE ε4-**	**FH+ ApoE ε4+**
**Drusen number**	**FH- ApoE ε4-**		0,838	0,311	0,769
	**FH- ApoE ε4+**	0,645		0.774	0,951
	**FH+ ApoE ε4-**	0,129	0,275		0,628
	**FH+ ApoE ε4+**	0,172	0,340	0,802	

(FH: Familiar history; ApoE: apolipoprotein E)

Table S2. P- value of drusen number and size groups characterized by history family, ApoE ε4 genotype and diabetes mellitus. Mann Whitney U test.

		Drusen size			
		**FH- ApoE ε4- DM-**	**FH- ApoE ε4+ DM-**	**FH+ ApoE ε4- DM-**	**FH+ ApoE ε4+ DM-**
**Drusen number**	**FH- ApoE ε4- DM-**		0,811	0,372	0,843
	**FH- ApoE ε4+ DM-**	0,540		0,861	0,932
	**FH+ ApoE ε4- DM-**	0,220	0,295		0,552
	**FH+ ApoE ε4+ DM-**	0,222	0,335	0,904	

(FH: Familiar history; ApoE: apolipoprotein E; DM: Diabetes Mellitus)
